# Economic and epidemiological impact of youth suicide in countries with the highest human development index

**DOI:** 10.1371/journal.pone.0232940

**Published:** 2020-05-19

**Authors:** Christopher M. Doran, Irina Kinchin

**Affiliations:** 1 Cluster for Resilience and Wellbeing, Centre for Indigenous Health Equity Research, School of Health, Medical and Applied Sciences, Central Queensland University Australia, Brisbane, Australia; 2 Centre for Health Economics Research and Evaluation and the Centre for Improving Palliative, Aged and Chronic Care Through Clinical Research and Translation, University of Technology Sydney, Ultimo, Australia; 3 Global Brain Health Institute, Dublin, Ireland; University of North Texas Health Science Center, UNITED STATES

## Abstract

This research estimates the economic and epidemiological impact of youth suicide in countries with the highest human development index. The study relied on secondary analysis of suicide mortality data for youth aged between 15–24 years in countries with the highest human development index–Norway, Australia, Switzerland, Germany, Denmark, Singapore, Netherlands, Ireland, Canada and the United States. The impact of youth suicide is measured using years of life lost, years of productive life lost and present economic value of lost productivity. Costs are expressed in 2014 International dollars. Future earning potential is estimated using adjusted gross domestic product per capita, employment potential and historical trends in productivity and real interest rates. In 2014, an estimated 6,912 young people living in the most developed countries in the world lost their lives to suicide. These preventable deaths resulted in a loss of 406,730 years of life at a cost of $5.53 billion in lost economic income with the average cost of suicide estimated at $802,939. The United States stands out as a country with the most significant youth suicide problem accounting for 77% of total costs. Reducing youth suicide requires a multifaceted approach and significant investment by governments.

## Introduction

Globally, suicide is the second most common cause of death, after road traffic accidents, among young people aged between 15–24 years [1]. A growing evidence base suggests that engaging in self-harm is the strongest predictor of future suicidal behaviour [2]. However, limited data exist on the extent of suicide attempts and suicidal ideation [2]. In Australia, data suggest that for every suicide, 129 people think about ending their life, 32 make a plan to suicide, and 23 attempt suicide each and every year [3].

Suicides are preventable [1]. In a recent review of suicide prevention strategies, Zalsman et al (2017) find that there is now strong evidence to suggest that restricting access to lethal means can prevent suicide and that school-based awareness programmes play a role in reducing suicide attempts and ideation. However, the authors also note that in the quest for effective suicide prevention initiatives, no single strategy clearly stands above the others and data suggest that each specific risk group might need a tailored preventive approach [4].

Adolescence and young adulthood is a clear example of a vulnerable group that requires special attention [5, 6]. Evidence suggests that risks of suicidal behaviour increase among this age group with epidemiological data clearly showing that suicide is a now a major cause of death and disability. More needs to be done and it has been suggested that the implementation of proven, evidence-based, and cost-effective strategies are the duty and responsibility of public health policy makers and health-care providers [4].

In addition to the accumulating evidence on youth suicide and self-harm morbidity and premature mortality [1, 6], there is a growing recognition of making an economic case for investing in youth suicide prevention [7]. Economic evidence can play a pivotal role in health policy in low, middle and high income country contexts [7]. Such evidence can assist public health-care decision-makers to understand the magnitude of adverse outcomes associated with suicide and the potential benefits to be achieved by investing in effective strategies to address suicidal behaviour. Although there has been several attempts at costing suicide at the population or workplace level [8–15], there is a paucity of research examining the economic impact of youth suicide [8, 16, 17]. Weinstein and Saturno (1989) reported the economic cost of youth suicide in the United States at $USD2.26 billion [17], Platt et al (2006) calculated the lifetime costs of completed suicide for young people in Scotland at £9.75 million [8]; Kinchin and Doran (2018) estimated the total economic loss of youth suicide in Australia at $AUD511 million [16].

A number of socio-economic indicators, including suicide rates, alcohol consumption and divorce rates, are known to be more common in more equal [income] countries [18]. Yet, these trends are not well known and largely unanalysed. The current study aims to fill this void by attempting to quantify and compare the economic and epidemiological impact of youth suicide in countries with the highest human development index.

## Materials and methods

### Countries with the highest human development

The United Nations Development Programme publishes the Human Development Report as an independent intellectual exercise that has become an important tool for raising awareness about human development around the world [19]. The composite Human Development Index integrates three basic dimensions of human development: life expectancy at birth; mean years of schooling; and gross national income per capita. Life expectancy at birth estimates the number of years a newborn infant could expect to live if prevailing patterns of age-specific mortality rates at the time of birth stay the same throughout the infant’s life. Mean years of schooling estimate the average number of years of education received by people ages 25 and older, converted from education attainment levels using official durations of each level. Gross national income per capita estimates the aggregate income of an economy converted to international dollars using purchasing power parity rates divided by midyear population [19]. International dollars are an accepted tool to compare standards of living across countries.

### Country level suicide data

World Health Organisation (WHO) country level data on crude suicide mortality rates (per 100,000) for youth aged between 15–24 years [6] are combined with population estimates [20] to estimate the number of youth suicide deaths by country.

### Economic and epidemiological considerations

The impact of youth suicide is measured using years of life lost (YLL), years of productive life lost (YPLL) and the economic value of lost productivity.

#### Years of life lost (YLL)

In the absence of country-specific age data on suicide mortality rates, the average age of a death by suicide is assumed to be 19.5 years, the mid-point of the 15–24 age bracket. For each country, this age was subtracted from the life expectancy at birth to obtain an estimate of average YLL.

#### Years of productive life lost (YPLL)

YPLL was derived by subtracting the average age of a death by suicide (i.e. 20 years) from the retirement age in each country [21]. However, not all persons who die by suicide may be productive members of society. Evidence suggests that a mental illness reduces an individual’s capacity to work, whether temporarily or permanently [22, 23], with economic output correspondingly reduced [24, 25]. To account for the fact that not all young people who died by suicide would have been employed, the probability of employment within each country [26] was reduced by 8.6 percentage points, consistent with data reported by the Centre for Mental Health [27].

#### Economic value of lost economic productivity

The economic value of lost economic productivity is calculated using the human capital method that combines the present value of average earnings foregone with the number of people who die by suicide together with the adjusted employment rate. Gross domestic product (GDP) per capita (expressed in 2014 current international dollars) is used as a proxy for economic value [20].

GDP per capita was adjusted to reflect income gender disparity using data on the gender wage gap. According to the Organization for Economic Cooperation and Development (OECD), the average gender wage gap for developed nations was 13% in 2017 [28], i.e., male wages were 13% higher than female wages.

Economic output changes over time. Historical patterns are often seen as a useful proxy for future patterns. To account for future growth in earning potential, a productivity factor, using GDP per capita as a proxy, is applied to future earning potential. The average growth in GDP per capita for each country, over the period 2000–2018 (inclusive) [20] is applied to adjusted GDP per capita.

It is also widely acknowledged that a dollar today is worth more than a dollar tomorrow due to the notion of time preference [29]. Future earning potential is converted to present value dollars using a discount rate that considers this time preference. The average change in real interest rates (i.e., adjusted for inflation) for each country, over the period 2000–2018 (inclusive) [20, 30], is used to discount future earnings to present value dollars.

### Sensitivity analysis

Given the range of data sources and assumptions used in the analysis, several univariate sensitivity analyses have been undertaken to test the robustness of results to variations in key parameters. These analyses explored variations in three key variables–GDP per capita estimates, the productivity factor and the discount rate. Sensitivity analysis 1 replaced adjusted GDP per capita with unadjusted GDP per capita. Sensitivity analysis 2 applied a productivity factor of 0% and a discount rate of 1%. Sensitivity analysis 3 applied a productivity factor of 0% and a discount rate of 3%. Sensitivity analysis 4 applied a productivity factor of 0% and a discount rate of 5%. Sensitivity analysis 5 replaced adjusted GDP per capita with unadjusted GDP per capita, applied a productivity factor of growth rate of 0% and a discount rate of 3%.

## Results

### Human development index scores

The countries with the highest human development index are listed in Table 1 together with the human development index score [19], life expectancy at birth [20], GDP per capita (in 2014 International dollars) and adjusted GDP per capita to reflect gender inequality in wages rates. From a maximum of one, Norway has the highest human development index score at 0.949, followed by Australia and Switzerland (0.939), Germany (0.926), Denmark and Singapore (0.925), Netherlands (0.924), Ireland (0.923), Canada and the United States (0.92). Male life expectancy is highest in Switzerland at 81.1 years followed by Australia at 80.7 years. Female life expectancy is highest in Singapore at 85.9 years followed by Switzerland at 85.1 years. Singapore has the highest GDP per capita at $86,612 followed by Norway at $66,015. The adjusted GDP per capita reflects the average gender wage gap across developed nations [28]. Singapore has the widest range with male GDP per capita estimated at $98,263 and female GDP per capita estimated at $74,960.

**Table 1:** Selective indicators for top ranking countries according to human development index.

Country	Human development index score	Life expectancy at birth (years)		GDP per capita (2014 International dollars)	Adjusted GDP per capita	
		Males	Females		Males	Females
Norway	0.949	79.6	83.6	$66,015	$74,896	$57,135
Australia	0.939	80.7	84.6	$46,880	$53,187	$40,574
Switzerland	0.939	81.1	85.1	$61,902	$70,230	$53,575
Germany	0.926	78.5	83.2	$47,191	$53,539	$40,842
Denmark	0.925	78.4	82.3	$47,901	$54,345	$41,457
Singapore	0.925	79.8	85.9	$86,612	$98,263	$74,960
Netherlands	0.924	79.8	83.5	$49,233	$55,856	$42,610
Ireland	0.923	79.1	83.3	$51,192	$58,079	$44,306
Canada	0.920	80.0	84.0	$45,646	$51,786	$39,505
United States	0.920	76.8	81.5	$55,033	$62,436	$47,630

Source: United National Human Development index [19]; World Bank indicators [20]; Organisation for Economic Co-operation and Development [28].

### Country level youth suicide data

Table 2 provides an overview of youth suicide for each country in the year 2014, the latest year available. No data exist for Iceland and is removed from further analysis. Australia has the highest crude suicide mortality rate among youth aged between 15–24 years at 11.58 per 100,000 persons, followed by the United States (11.5 per 100,000 persons) and Canada (11.23 per 100,000 persons). Denmark has the lowest crude suicide mortality rates among youth aged between 15–24 years at 5.24 per 100,000 persons. Crude suicide mortality rates are generally higher for males compared with females. The highest rates are seen in United States (17.99 per 100,000 males) followed by Australia (16.57 per 100,000 males). Australia has the highest crude suicide mortality rate in female youth aged between 15–24 years at 6.31 per 100,000, followed by Canada (6.08 per 100,000 females) and Singapore (6.02 per 100,000 females). Singapore has the lowest crude suicide mortality rate in young males (7.37 per 100,000) and Demark has the lowest crude suicide mortality rate in young females (1.98 per 100,000). The United States loses more lives to youth suicide than any other country with an estimated 5,120 deaths in 2014. Germany has the second highest number of youth suicide deaths at 521, followed by Canada at 519 deaths. Denmark loses the least to youth suicide with an estimated 39 deaths in 2014.

**Table 2:** Country level suicide data for youth aged between 15–24 years.

Country	Crude suicide mortality rates per 100,000			Population aged 15–24 years			Number of suicide deaths		
	Male	Female	Persons	Male	Female	Persons	Male	Female	Persons
Norway	11.04	5.23	8.22	347,445	327,942	675,387	38	17	55
Australia	16.57	6.31	11.58	1,620,853	1,530,577	3,151,430	269	97	365
Switzerland	10.83	3.69	7.34	486,011	465,673	951,684	53	17	70
Germany	8.92	2.87	5.98	4,495,948	4,223,785	8,719,733	401	121	521
Denmark	8.37	1.98	5.24	380,498	359,706	740,204	32	7	39
Singapore	7.37	6.02	6.71	370,874	357,824	728,698	27	22	49
Netherlands	8.41	3.34	5.92	1,056,637	1,012,302	2,068,939	89	34	123
Ireland	16.11	3.46	9.94	263,688	257,182	520,870	42	9	52
Canada	16.17	6.08	11.23	2,361,878	2,256,361	4,618,239	382	137	519
United States	17.99	4.62	11.50	22,760,623	21,748,724	44,509,347	4,094	1,005	5,120

*Source*: WHO mortality database [6]; World Bank indicators [20].

### Key parameters used to estimate the economic value of lost economic productivity

Table 3 provides an overview of the key parameters used to estimate lost economic productivity: retirement age, GDP per capita (used as a proxy for productivity growth), the real interest rate (used as a proxy for the discount rate) and the adjusted employment rate (used to reflect reduced economic potential for a person with a mental illness). The retirement age is consistent across countries at around 65 to 66 years with Norway and Ireland the exception with a retirement age of 62 and 67 years, respectively. There is no variation in retirement age by gender except for Switzerland where the retirement age for females is one year earlier than males. Over the period 2000–2018, the average growth in GDP per capita was the highest in Ireland at 3.7% followed by Singapore at 3.3%. Growth was lowest in Denmark and Norway at 0.8%. Over the period 2000–2018, average real interest rates were the highest in Norway at 4.2% followed by Singapore at 4.1%. Average real interest rates were the lowest in Canada at 1.9%. Singapore had the highest employment rates at an estimated 100%, adjusted to 91.4% to reflect reduced earning potential due to possible mental illness. Most countries had adjusted male employment rates ranging from 44% to 53%, with slightly lower rates for females.

**Table 3:** Key parameters used to estimate lost economic productivity.

Country	Retirement age (years		GDP per capita growth %	Real interest rate %	Adjusted employment rate %*	
	Males	Females			Males	Females
Norway	62	62	0.8%	4.2%	44.1%	38.7%
Australia	66	66	1.5%	3.8%	45.5%	37.3%
Switzerland	65	64	1.0%	2.6%	45.0%	37.8%
Germany	66	66	1.4%	2.0%	44.8%	38.0%
Denmark	65	65	0.8%	2.9%	44.2%	38.6%
Singapore	62	62	3.3%	4.1%	91.4%	91.4%
Netherlands	66	66	1.1%	2.0%	45.5%	37.3%
Ireland	67	67	3.7%	2.0%	45.7%	37.1%
Canada	65	65	1.1%	1.9%	52.4%	47.6%
United States	66	66	1.2%	2.8%	53.1%	46.9%

Source: World Bank indicators [20]; Trading economics [30]

*actual employment rate [20] adjusted downward by 8.6 percentage points [27].

### Average economic and epidemiological considerations of youth suicide

Table 4 provides an overview of the average economic and epidemiological consequences of youth suicide, by country and gender. The average number of years of life lost (YLL) are relatively consistent across countries (due to similarities in life expectancies) ranging from a high of 66.4 years in Switzerland females to 57.3 years in United States males. YLL are generally higher for females than males. The United States had the lowest YLL in both females (61.5 years) and males (56.8 years). The average number of years of productive life lost (YPLL) ranges from 42.5 to 47.5 years, reflecting similarities in the retirement age across countries (range 62 to 67 years). The present value of average earnings foregone differs by gender and country due to variations in adjusted GDP per capita, growth rates and real interest rates. Singapore and Switzerland have the highest present value of average earnings foregone at $2,134,632 and $1,928,023, respectively. Although Norway has the third highest level of adjusted GDP per capita, it has the third lowest present value of average earnings foregone in males at an estimated $1,555,266. due to the country’s low growth rate and high rate of interest.

**Table 4:** Average economic and epidemiological considerations of youth suicide.

Country	Average years of life lost		Average years of productive life lost		Present value of average earnings foregone	
	Males	Females	Males	Females	Males	Females
Norway	60.1	64.1	42.5	42.5	$1,555,266	$1,186,434
Australia	61.2	65.1	46.0	46.0	$1,222,619	$932,674
Switzerland	61.6	65.6	45.5	44.5	$1,928,023	$1,453,980
Germany	59.0	63.7	46.2	46.2	$1,666,833	$1,271,542
Denmark	58.9	62.8	45.5	45.5	$1,426,884	$1,088,497
Singapore	60.3	66.4	42.5	42.5	$2,134,632	$1,628,403
Netherlands	60.3	64.0	46.5	46.5	$1,734,299	$1,323,009
Ireland	59.6	63.8	47.5	47.5	$1,872,954	$1,428,782
Canada	60.5	64.5	45.5	45.5	$1,634,872	$1,247,161
United States	57.3	62.0	46.5	46.5	$1,681,155	$1,282,468

### Total economic and epidemiological considerations of youth suicide

Table 5 provides an overview of the total economic and epidemiological consequences of youth suicide, by country and gender. Also included in the table is an estimate of the mean cost of suicide per person per country, reflecting the present value of total earnings foregone (Table 4) divided by the total number of suicide deaths (Table 2). The burden of suicide in the United States far exceeds any other country at an estimated 296,893 YLL, 123,003 adjusted PYLL and a present value of total earnings foregone at $4.26 billion in (2014 International dollars). The countries with the next highest burden are Canada (31,941 YLL, 12,072 adjusted PYLL and lost earnings of $408 million, Germany (31,373 YLL, 10,418 adjusted PYLL and lost earnings of $357 million) and Australia (22,730 YLL, 7,280 adjusted PYLL and lost earnings of $183 million).The estimated average cost of suicide across all countries is estimated at $802,939, ranging from a low of $501.169 in Australia to a high of $1,747,170 in Singapore.

**Table 5:** Total average economic and epidemiological considerations of youth suicide.

Country	Total years of life lost			Years of adjusted productive years of life lost			Present value of total earnings foregone			Mean cost of suicide
	Males	Females	Persons	Males	Females	Persons	Males	Females	Persons	Persons
Norway	2,305	1,099	3,404	719	282	1,001	$26,305,543	$7,872,187	$34,177,730	$615,831
Australia	16,441	6,289	22,730	5,623	1,658	7,280	$149,441,334	$33,608,339	$183,049,672	$501,169
Switzerland	3,243	1,128	4,371	1,078	289	1,367	$45,679,731	$9,448,646	$55,128,377	$789,332
Germany	23,651	7,722	31,373	8,292	2,127	10,418	$299,341,057	$58,577,176	$357,918,232	$685,543
Denmark	1,875	447	2,321	640	125	765	$20,074,980	$2,987,618	$23,062,599	$592,241
Singapore	1,648	1,430	3,078	1,062	836	1,898	$53,333,292	$32,050,650	$85,383,942	$1,747,170
Netherlands	5,356	2,162	7,519	1,879	586	2,465	$70,094,965	$16,673,589	$86,768,554	$707,645
Ireland	2,531	568	3,099	922	157	1,079	$36,353,003	$4,716,626	$41,069,629	$799,496
Canada	23,099	8,842	31,941	9,103	2,969	12,072	$327,077,621	$81,383,123	$408,460,744	$787,182
United States	234,576	62,317	296,893	101,082	21,920	123,003	$3,654,521,777	$604,555,294	$4,259,077,072	$835,288
**Total**	**314,725**	**92,004**	**406,730**	**130,400**	**30,949**	**161,349**	**$4,682,223,303**	**$851,873,248**	**$5,534,096,551**	**$802,939**

### Sensitivity analysis

Results of the sensitivity analyses are provided in S1–S5 Tables. Compared to the baseline estimates (Tables 4 and 5), applying a lower discount rate of 1% combined with stable productivity growth (i.e., sensitivity analysis 2) had the largest impact, increasing the present value of total earnings foregone by $2.10 billion, from $5.53 billion to $7.64 billion. A lower opportunity cost of capital combined with stable annual growth results in substantially higher estimates of average earnings foregone that subsequently translate into higher total earnings foregone. Modifying GDP per capita (sensitivity analysis 1) had the smallest impact on reducing total earnings. A higher opportunity cost of capital (discount rate of 5%) combined with stable annual growth results (i.e., sensitivity analysis 4) reduces the present value of total earnings foregone by $1.70 billion, from $5.53 billion to $3.83 billion. The estimated average cost of suicide across all countries at baseline is estimated at $802,939, varying in the sensitivity analysis from a low of $555,713 (sensitivity analysis 4) to a high of $1,108,260 (sensitivity analysis 2).

## Discussion

This study has attempted to quantify the economic and epidemiological impact of youth suicide in countries with the highest human development index. This is the first study of its kind and before considering the main findings, it is important to reflect on potential strengths and limitations. First, the analysis is dependent on the quality and timing of data. The quality of suicide mortality data is questionable. Although the quality of vital registration systems is likely to be high in the developed countries included in this analysis, under-reporting and misclassification are greater problems for suicide than for most other causes of death. Suicide registration is a complicated, multilevel procedure that includes medical and legal concerns and involves several responsible authorities that can vary from country to country [1]. Second, due to data availability, the analysis has been restricted to 2014, the latest year available across all countries in the analysis. Changes may have occurred in suicide rates since 2014 that may render these estimates out of date. Third, various assumptions have been made in quantifying the economic and epidemiological impact of youth suicide. GDP per capita is used as an income proxy and has been adjusted to reflect income disparity. OECD data has been used as the source for this adjustment and may not reflect income disparity in countries outside of the OECD, this may impact on gender estimates of forgone earnings but not necessarily on overall estimates. Future earning potential also relies on historical trends in productivity and real interest rates. As noted in the sensitivity analyses, changes in the discount rate have significant impacts on overall values. Nevertheless, by taking an average of historical trends in growth and real interest rates, an effort has been made to predict the future based on the past. Finally, the analysis is limited to fatality by suicide with a focus on potential earnings foregone. A more accurate assessment of the impact of suicidal behaviour would take into account preventive efforts together with the cost of self-harming behaviour [16, 31].

As the data in these analyses demonstrate, even the most developed nations are not immune from the impact of suicide. In 2014, an estimated 6,912 young people living in the most developed countries in the world lost their lives to suicide. These preventable deaths resulted in a loss of 406,730 years of life and 161,349 years of productive life, adjusted for employment potential. The present value of lost earning potential due to youth suicide in these countries is estimated at US$5.534 billion. The burden of suicide in the United States far exceeds any other country at an estimated 296,893 YLL, 123,003 adjusted PYLL and a present value of total earnings foregone at $4.26 billion in (2014 International dollars). The countries with the next highest burden are Canada (31,941 YLL, 12,072 adjusted PYLL and lost earnings of $408 million, Germany (31,373 YLL, 10,418 adjusted PYLL and lost earnings of $357 million) and Australia (22,730 YLL, 7,280 adjusted PYLL and lost earnings of $183 million).

The estimated average cost of suicide across all countries is estimated at $802,939, ranging from $501.169 in Australia to $1,747,170 in Singapore. As noted earlier, variations in cost per suicide per country reflect variations in adjusted GDP per capita, growth rates and real interest rates. For example, out of all the countries examined, Singapore has the highest GDP per capita ($86,612 per person), one of the highest annual growth rates (3.3%) and one of the highest real interest rates (4.1%). The estimated average cost of suicide across all countries at baseline is estimated at $802,939, varying in the sensitivity analysis from $555,713 (sensitivity analysis 4) to a high of $1,108,260 (sensitivity analysis 2). This range confirm the sensitivity of the results to underlying assumptions with lower interest and growth rates, having relatively greater impact on estimates of present value of total earnings foregone and subsequent cost per suicide.

The United States stands out as a country with the most significant youth suicide problem. It has one of the highest crude suicide mortality rates (both male and female) and the greatest number of youth suicide deaths of any country– 5,120. The present value of lost earning potential due to youth suicide in the United States is estimated at US$4.26 billion, representing 77% of total value from the top ten ranked countries. Modifying the discount rate has the largest impact on present value of average and total earnings foregone.

Youth suicide rates are unacceptable, particularly given suicide is preventable. The World Health Organisation’s mental health action plan has set the goal of reducing the rate of suicide in countries by 10% by 2020 [32]. Reducing youth suicide requires a multifaceted approach and the WHO suicide prevention framework provides a platform to enable change [33]. Governments, international organizations, non-governmental organizations and local communities all have a part to play in combating suicide. A systems-based approach to suicide prevention was recently proposed in Australia that builds on nine strategies, including aftercare and crisis care; psychological and pharmacotherapy treatments; building the capacity and support of general practice teams; frontline staff training; gatekeeper training; school programs; community campaigns; media guidelines; and means restriction, which when implemented within a specific community at the same time are likely to lead to suicide reduction [34]. Although the effectiveness of this approach is yet to be established, our findings suggest that the impact of meeting a 10% reduction in youth suicide in the countries examined in this research would avert 691 deaths (512 in the United States), 40,673 years of life lost and save $553 million in foregone earnings. These savings would more than offset any investment in suicide prevention.

Although there is a lack of robust economic studies that assess the cost-effectiveness or return on investment of suicide prevention strategies [1], the evidence base in increasing. A WHO review of suicide prevention strategies that included cost as a parameter of interest showed that two thirds of the strategies assessed as being effective or promising were categorized as low-cost and that low cost was also closely associated with universal or selective prevention approaches [35]. A recent article by Kinchin et al (2019) modelled the potential return on investment (ROI) on a population basis of implementing a suicide education and awareness training in schools. From a societal perspective, every dollar invested in training resulted in a ROI of $31.21 [36]. Doran et al (2015) examined the potential impact of introducing a multi-faceted strategy called Mates in Construction to address suicide prevention in the work place in the Australian state of New South Wales. The authors report that with a budget of rolling out the MIC program in New South Wales at $AUD800,000 each year, the benefit cost ratio is equivalent to 4.6:1, representing a positive economic investment of public funds [14]. More research is needed to contribute to this evidence base.

## Conclusion

Economic evidence can assist public health-care decision-makers to understand the magnitude of adverse outcomes associated with suicide and the potential benefits to be achieved by investing in effective strategies to address suicidal behaviour. This research has attempted to quantify the economic and epidemiological impact of youth suicide in countries with the highest human development index. The results are staggering–almost 7,000 young lives are lost each year to suicide representing a loss of 406,730 years of life at a cost of over $5.53 billion. Reducing youth suicide requires a multifaceted approach and significant investment by governments.

## Supporting information

S1 Table Sensitivity analysis 1. (DOCX)

S2 Table Sensitivity analysis 2. (DOCX)

S3 Table Sensitivity analysis 3. (DOCX)

S4 Table Sensitivity analysis 4. (DOCX)

S5 Table Sensitivity analysis 5. (DOCX)
