# Supplementary material for: Economic and epidemiological impact of youth suicide in countries with the highest human development index
Source: PLoS One. 2020 May 19;15(5):e0232940. doi: 10.1371/journal.pone.0232940 (PMC7236997; doi:10.1371/journal.pone.0232940)
Supplement: S1 Table — (DOCX) [file pone.0232940.s001.docx]

S1 Table: Sensitivity analysis 1: Replaced adjusted GDP per capita with unadjusted GDP per capita

| **Country** | **Number of suicide deaths** | | **Adjusted employment rate** | | **Present value of average earnings foregone** | | **Present value of total earnings foregone** | | | **Mean cost of suicide** |
| --- | --- | --- | --- | --- | --- | --- | --- | --- | --- | --- |
|  | **Male** | **Female** | **Male** | **Female** | **Male** | **Female** | **Male** | **Female** | **Persons** | **Persons** |
| Norway | 38 | 17 | 44% | 39% | $1,370,850 | $1,370,850 | $23,186,356 | $9,095,819 | $32,282,175 | $581,676 |
| Australia | 269 | 97 | 46% | 37% | $1,077,646 | $1,077,646 | $131,721,282 | $38,832,330 | $170,553,613 | $466,956 |
| Switzerland | 53 | 17 | 45% | 38% | $1,699,407 | $1,679,983 | $40,263,243 | $10,917,319 | $51,180,562 | $732,807 |
| Germany | 401 | 121 | 45% | 38% | $1,469,188 | $1,469,188 | $263,846,600 | $67,682,258 | $331,528,858 | $634,998 |
| Denmark | 32 | 7 | 44% | 39% | $1,257,691 | $1,257,691 | $17,694,584 | $3,452,006 | $21,146,589 | $543,039 |
| Singapore | 27 | 22 | 91% | 91% | $1,881,517 | $1,881,517 | $47,009,281 | $37,032,518 | $84,041,799 | $1,719,707 |
| Netherlands | 89 | 34 | 46% | 37% | $1,528,654 | $1,528,654 | $61,783,433 | $19,265,288 | $81,048,721 | $660,997 |
| Ireland | 42 | 9 | 46% | 37% | $1,650,868 | $1,650,868 | $32,042,435 | $5,449,766 | $37,492,201 | $729,855 |
| Canada | 382 | 137 | 52% | 48% | $1,441,016 | $1,441,016 | $288,294,293 | $94,033,102 | $382,327,395 | $736,818 |
| United States | 4094 | 1005 | 53% | 47% | $1,481,811 | $1,481,811 | $3,221,185,753 | $698,525,778 | $3,919,711,532 | $768,732 |
| ***Total*** | ***5427*** | ***1466*** |  |  | ***$14,858,648*** | ***$14,839,225*** | ***$4,127,027,260*** | ***$984,286,183*** | ***$5,111,313,444*** | ***$741,598*** |
