# Supplementary material for: Economic and epidemiological impact of youth suicide in countries with the highest human development index
Source: PLoS One. 2020 May 19;15(5):e0232940. doi: 10.1371/journal.pone.0232940 (PMC7236997; doi:10.1371/journal.pone.0232940)
Supplement: S2 Table — (DOCX) [file pone.0232940.s002.docx]

S2 Table: Sensitivity analysis 2: Applied a productivity factor of 0% and a discount rate of 1%

| **Country** | **Number of suicide deaths** | | **Adjusted employment rate** | | **Present value of average earnings foregone** | | **Present value of total earnings foregone** | | | **Mean cost of suicide** |
| --- | --- | --- | --- | --- | --- | --- | --- | --- | --- | --- |
|  | **Male** | **Female** | **Male** | **Female** | **Male** | **Female** | **Male** | **Female** | **Persons** | **Persons** |
| Norway | 38 | 17 | 44% | 39% | $2,632,968 | $2,008,558 | $44,533,633 | $13,327,118 | $57,860,751 | $1,042,563 |
| Australia | 269 | 97 | 46% | 37% | $2,006,594 | $1,530,729 | $245,267,024 | $55,158,884 | $300,425,908 | $822,531 |
| Switzerland | 53 | 17 | 45% | 38% | $2,604,913 | $1,952,917 | $61,716,963 | $12,690,972 | $74,407,935 | $1,065,379 |
| Germany | 401 | 121 | 45% | 38% | $2,019,886 | $1,540,869 | $362,744,684 | $70,984,446 | $433,729,130 | $830,749 |
| Denmark | 32 | 7 | 44% | 39% | $2,015,747 | $1,537,711 | $28,359,760 | $4,220,584 | $32,580,344 | $836,654 |
| Singapore | 27 | 22 | 91% | 91% | $3,454,424 | $2,635,205 | $86,308,004 | $51,866,807 | $138,174,811 | $2,827,404 |
| Netherlands | 89 | 34 | 46% | 37% | $2,107,133 | $1,607,425 | $85,163,730 | $20,258,018 | $105,421,748 | $859,772 |
| Ireland | 42 | 9 | 46% | 37% | $2,227,370 | $1,699,148 | $43,232,019 | $5,609,145 | $48,841,163 | $950,783 |
| Canada | 382 | 137 | 52% | 48% | $1,920,817 | $1,465,294 | $384,284,695 | $95,617,329 | $479,902,024 | $924,863 |
| United States | 4094 | 1005 | 53% | 47% | $2,355,356 | $1,796,782 | $5,120,112,291 | $847,003,023 | $5,967,115,314 | $1,170,267 |
| ***Total*** | ***5427*** | ***1466*** |  |  | ***$23,345,207*** | ***$17,774,638*** | ***$6,461,722,803*** | ***$1,176,736,326*** | ***$7,638,459,129*** | ***$1,108,260*** |
