# Supplementary material for: Economic and epidemiological impact of youth suicide in countries with the highest human development index
Source: PLoS One. 2020 May 19;15(5):e0232940. doi: 10.1371/journal.pone.0232940 (PMC7236997; doi:10.1371/journal.pone.0232940)
Supplement: S3 Table — (DOCX) [file pone.0232940.s003.docx]

S3 Table: Sensitivity analysis 3: Applied a productivity factor of 0% and a discount rate of 3%

| **Country** | **Number of suicide deaths** | | **Adjusted employment rate** | | **Present value of average earnings foregone** | | **Present value of total earnings foregone** | | | **Mean cost of suicide** |
| --- | --- | --- | --- | --- | --- | --- | --- | --- | --- | --- |
|  | **Male** | **Female** | **Male** | **Female** | **Male** | **Female** | **Male** | **Female** | **Persons** | **Persons** |
| Norway | 38 | 17 | 44% | 39% | $1,849,725 | $1,411,062 | $31,285,979 | $9,362,630 | $40,648,609 | $732,427 |
| Australia | 269 | 97 | 46% | 37% | $1,370,915 | $1,045,802 | $167,567,740 | $37,684,844 | $205,252,584 | $561,958 |
| Switzerland | 53 | 17 | 45% | 38% | $1,791,900 | $1,352,777 | $42,454,642 | $8,790,976 | $51,245,618 | $733,739 |
| Germany | 401 | 121 | 45% | 38% | $1,379,997 | $1,052,730 | $247,829,104 | $48,496,953 | $296,326,057 | $567,572 |
| Denmark | 32 | 7 | 44% | 39% | $1,386,617 | $1,057,780 | $19,508,469 | $2,903,308 | $22,411,777 | $575,528 |
| Singapore | 27 | 22 | 91% | 91% | $2,426,818 | $1,851,297 | $60,633,508 | $36,437,715 | $97,071,224 | $1,986,321 |
| Netherlands | 89 | 34 | 46% | 37% | $1,439,515 | $1,098,133 | $58,180,706 | $13,839,527 | $72,020,233 | $587,365 |
| Ireland | 42 | 9 | 46% | 37% | $1,511,282 | $1,152,881 | $29,333,147 | $3,805,834 | $33,138,981 | $645,111 |
| Canada | 382 | 137 | 52% | 48% | $1,321,316 | $1,007,965 | $264,346,597 | $65,774,453 | $330,121,050 | $636,207 |
| United States | 4094 | 1005 | 53% | 47% | $1,609,092 | $1,227,495 | $3,497,871,041 | $578,641,088 | $4,076,512,129 | $799,483 |
| ***Total*** | ***5427*** | ***1466*** |  |  | ***$16,087,178*** | ***$12,257,921*** | ***$4,419,010,933*** | ***$805,737,329*** | ***$5,224,748,261*** | ***$758,056*** |
