# Supplementary material for: Economic and epidemiological impact of youth suicide in countries with the highest human development index
Source: PLoS One. 2020 May 19;15(5):e0232940. doi: 10.1371/journal.pone.0232940 (PMC7236997; doi:10.1371/journal.pone.0232940)
Supplement: S4 Table — (DOCX) [file pone.0232940.s004.docx]

S4 Table: Sensitivity analysis 4: Applied a productivity factor of 0% and a discount rate of 5%

| **Country** | **Number of suicide deaths** | | **Adjusted employment rate** | | **Present value of average earnings foregone** | | **Present value of total earnings foregone** | | | **Mean cost of suicide** |
| --- | --- | --- | --- | --- | --- | --- | --- | --- | --- | --- |
|  | **Male** | **Female** | **Male** | **Female** | **Male** | **Female** | **Male** | **Female** | **Persons** | **Persons** |
| Norway | 38 | 17 | 44% | 39% | $1,379,600 | $1,052,427 | $23,334,356 | $6,983,030 | $30,317,386 | $546,274 |
| Australia | 269 | 97 | 46% | 37% | $1,004,172 | $766,032 | $122,740,444 | $27,603,490 | $150,343,935 | $411,624 |
| Switzerland | 53 | 17 | 45% | 38% | $1,318,311 | $999,703 | $31,234,107 | $6,496,536 | $37,730,643 | $540,230 |
| Germany | 401 | 121 | 45% | 38% | $1,010,824 | $771,106 | $181,530,493 | $35,523,171 | $217,053,664 | $415,737 |
| Denmark | 32 | 7 | 44% | 39% | $1,020,142 | $778,215 | $14,352,485 | $2,135,979 | $16,488,464 | $423,419 |
| Singapore | 27 | 22 | 91% | 91% | $1,810,020 | $1,380,773 | $45,222,937 | $27,176,730 | $72,399,668 | $1,481,479 |
| Netherlands | 89 | 34 | 46% | 37% | $1,054,432 | $804,373 | $42,616,836 | $10,137,328 | $52,754,164 | $430,239 |
| Ireland | 42 | 9 | 46% | 37% | $1,102,262 | $840,860 | $21,394,287 | $2,775,805 | $24,170,093 | $470,515 |
| Canada | 382 | 137 | 52% | 48% | $972,099 | $741,565 | $194,481,203 | $48,390,617 | $242,871,820 | $468,061 |
| United States | 4094 | 1005 | 53% | 47% | $1,178,645 | $899,129 | $2,562,158,649 | $423,849,322 | $2,986,007,971 | $585,614 |
| ***Total*** | ***5427*** | ***1466*** |  |  | ***$11,850,505*** | ***$9,034,181*** | ***$3,239,065,799*** | ***$591,072,009*** | ***$3,830,137,808*** | ***$555,713*** |
