# Supplementary material for: Economic and epidemiological impact of youth suicide in countries with the highest human development index
Source: PLoS One. 2020 May 19;15(5):e0232940. doi: 10.1371/journal.pone.0232940 (PMC7236997; doi:10.1371/journal.pone.0232940)
Supplement: S5 Table — (DOCX) [file pone.0232940.s005.docx]

S5 Table: Sensitivity analysis 5: Replaced adjusted GDP per capita with unadjusted GDP per capita, applied a productivity factor of growth rate of 0% and a discount rate of 3%.

| **Country** | **Number of suicide deaths** | | **Adjusted employment rate** | | **Present value of average earnings foregone** | | **Present value of total earnings foregone** | | | **Mean cost of suicide** |
| --- | --- | --- | --- | --- | --- | --- | --- | --- | --- | --- |
|  | **Male** | **Female** | **Male** | **Female** | **Male** | **Female** | **Male** | **Female** | **Persons** | **Persons** |
| Norway | 38 | 17 | 44% | 39% | $1,630,393 | $1,630,393 | $27,576,235 | $10,817,933 | $38,394,167 | $691,805 |
| Australia | 269 | 97 | 46% | 37% | $1,208,359 | $1,208,359 | $147,698,345 | $43,542,477 | $191,240,822 | $523,595 |
| Switzerland | 53 | 17 | 45% | 38% | $1,579,425 | $1,563,049 | $37,420,570 | $10,157,422 | $47,577,993 | $681,225 |
| Germany | 401 | 121 | 45% | 38% | $1,216,363 | $1,216,363 | $218,442,693 | $56,035,191 | $274,477,884 | $525,725 |
| Denmark | 32 | 7 | 44% | 39% | $1,222,199 | $1,222,199 | $17,195,246 | $3,354,591 | $20,549,837 | $527,714 |
| Singapore | 27 | 22 | 91% | 91% | $2,139,058 | $2,139,058 | $53,443,872 | $42,101,498 | $95,545,370 | $1,955,099 |
| Netherlands | 89 | 34 | 46% | 37% | $1,268,824 | $1,268,824 | $51,281,911 | $15,990,707 | $67,272,618 | $548,645 |
| Ireland | 42 | 9 | 46% | 37% | $1,332,081 | $1,332,081 | $25,854,960 | $4,397,402 | $30,252,363 | $588,918 |
| Canada | 382 | 137 | 52% | 48% | $1,164,640 | $1,164,640 | $233,001,619 | $75,998,261 | $308,999,880 | $595,502 |
| United States | 4094 | 1005 | 53% | 47% | $1,418,293 | $1,418,293 | $3,083,109,925 | $668,583,536 | $3,751,693,461 | $735,780 |
| ***Total*** | ***5427*** | ***1466*** |  |  | ***$14,179,636*** | ***$14,163,260*** | ***$3,895,025,377*** | ***$930,979,018*** | ***$4,826,004,395*** | ***$700,202*** |
